# How vertical hand movements impact brain activity elicited by literally and metaphorically related words: an ERP study of embodied metaphor

**DOI:** 10.3389/fnhum.2014.01031

**Published:** 2014-12-23

**Authors:** Megan Bardolph, Seana Coulson

**Affiliations:** Cognitive Science Department, University of CaliforniaSan Diego, La Jolla, CA, USA

**Keywords:** semantics, embodiment, grounded meaning, N400, LPC, motor resonance, compatibility effects

## Abstract

Embodied metaphor theory suggests abstract concepts are metaphorically linked to more experientially basic ones and recruit sensorimotor cortex for their comprehension. To test whether words associated with spatial attributes reactivate traces in sensorimotor cortex, we recorded EEG from the scalp of healthy adults as they read words while performing a concurrent task involving either upward- or downward- directed arm movements. ERPs were time-locked to words associated with vertical space—either literally (ascend, descend) or metaphorically (inspire, defeat)—as participants made vertical movements that were either congruent or incongruent with the words. Congruency effects emerged 200–300 ms after word onset for literal words, but not until after 500 ms post-onset for metaphorically related words. Results argue against a strong version of embodied metaphor theory, but support a role for sensorimotor simulation in concrete language.

## Introduction

Embodied or grounded theories of cognition suggest the neural substrate of word meaning involves brain regions extending considerably beyond the traditional language areas in the brain. Because words are frequently encountered in the context of the objects, actions, and events they represent, their linguistic representations are associated with experiential traces of their referents. The word “dog,” for example, is associated with the perceptual attributes of dogs, motoric routines for interacting with dogs, as well as the features of situations in which one typically encounters dogs. According to embodied models of language comprehension, understanding the meaning of “dog” involves the reactivation of contextually relevant experiential traces in sensorimotor cortex (e.g., Zwaan and Madden, 2005).

Embodied models of language meaning contrast with symbolic approaches prevalent in the Twentieth century (e.g., Pylyshyn, 1984), but accord better with views advanced by Nineteenth century neurologists. Observations of patients with brain damage led these neurologists to suggest that concepts are represented in a distributed array of brain regions that includes sensory and motor areas (Wernicke, 1874/1977; Head, 1926). Recent years have provided evidence in support of embodied meaning as behavioral research has shown tight links between the language system and both perception (Barsalou, 2010) and action (Fischer and Zwaan, 2008), and neuroimaging studies have shown that linguistic stimuli activate brain regions associated with sensorimotor processing (Pulvermüller, 2005; Barsalou, 2008). However, the precise import of these sensorimotor activations is controversial, as is the issue of whether amodal representations play a significant role in the neural representation of meaning (see Meteyard et al., 2012 for an insightful review).

Although there is growing agreement that the neural representation of concrete concepts involves sensory as well as motor processing regions, the representation of abstract concepts is hotly contested. Perhaps the most radical suggestion to date is that of Gallese and Lakoff (2005) that embodied content underpins all conceptual structure. Their claim is that abstract concepts used in imagination and language comprehension recruit the same neural substrates as those recruited by primary experience involving direct perception and (inter) action. According to this model, abstract concepts are grounded in experience via the mediation of metaphoric mappings. The concept of time, for example, is grounded in experiences of motion through space such that neural resources recruited for our understanding of spatial motion can be redeployed for relevant inferences about the domain of time (Boroditsky, 2011). On such accounts, metaphoric reference to time *passing, creeping*, or *flying* are understandable because of an overlap in the neural substrate of concepts for time and for motion (Lakoff and Johnson, 1999).

Indeed, metaphor theorists have noted that spatial metaphors are highly prevalent, being a common feature of languages throughout the world (Kovecses, 2006), and applying to numerous abstract domains (Lakoff and Johnson, 1980). Orientation metaphors, for example, occur in the domains of morality, health, rationality, consciousness, and control, consistently mapping positive elements upwards and negative ones downwards. In the domain of morality, for example, we talk about *upstanding citizens* vs. people of *low character*; in the domain of control, we talk about the *overlords* vs. the *underclass*. Similarly, positive emotions such as happiness are associated with upper regions of space, while negative ones such as sadness are associated with lower regions of space, as in “*I was feeling down, but having lunch with you has really cheered me up!*” Embodied metaphor theory suggests that spatial features are activated in understanding metaphoric uses of these words, just as they are for literal uses of “up,” “down,” “over,” and “under.” Moreover, because the theory posits links between the two domains in a metaphor, these spatial schemas are part of our concepts for morality, control, and emotions.

Consistent with embodied metaphor theory, behavioral research has shown that spatial attributes are active in judgment tasks involving abstract concepts structured by orientation metaphors. Much of the research on this topic has taken advantage of stimulus-response compatibility effects, or the finding that participants respond more quickly and accurately in judgment tasks when the nature of the response matches some feature of the stimulus (e.g., indicating the presence of a stimulus in the right side of space with a right hand response). For example, the action sentence compatibility effect (ACE) is the finding that participants respond more quickly to sentences about actions involving movement away from their bodies (“You closed the drawer.”) when the response requires a movement away from their bodies; likewise, participants respond more quickly to sentences about movement toward their bodies (“You opened the drawer.”) when the response involves movement toward their bodies (Glenberg and Kaschak, 2002). Santana and De Vega (2011) utilized the ACE paradigm to show that vertical motion verbs were subject to compatibility effects. In this study, participants read the Spanish equivalents of sentences such as “The pressured gas made the balloon rise,” (literal) and “His talent for politics made him rise to victory,” (metaphor), and pressed a button in response to the animated motion of the verb. Responses were faster when the direction of movement matched the direction of the verb, suggesting literal and metaphorical motion verbs activate movement schemas along a similar time course.

Some evidence supports the presence of compatibility effects even in the processing of individual words. For example, when asked to judge which of two social groups (such as “masters” and “servants”) was more powerful, participants responded faster when the more powerful group was presented at the top of the screen; when asked to judge which group was less powerful, participants' responses were faster when the chosen group (i.e., servants) was presented at the bottom of the screen (Schubert, 2005). Similarly, when asked to judge whether words (such as “hero” and “liar”) had a positive or a negative meaning, participants' responses were faster for positively valenced items when they appeared above the fixation point, and faster for negatively valenced items when they appeared below fixation (Meier and Robinson, 2004).

The observation of compatibility effects for both sentences and individual words is relevant because models of embodied meaning differ regarding the automaticity of motor activity during language comprehension, as well as the language processing stage at which such effects arise. Pulvermüller (2005) posits a strong embodiment model in which sensorimotor meaning activation is rooted in fundamental aspects of neuronal function. On this model, the frequent co-occurrence of words such as “raise” with, say, the action of raising one's hand, leads to the formation of neuronal ensembles connecting the neural representation of the word with the relevant motor programs. Once assembled, the acoustic representation of the word triggers the rapid, automatic activation of associated motor programs. Whereas strong embodiment models suggest the activation of motor schemas arises automatically in the course of word comprehension, weak embodiment models suggest sensorimotor activations are more relevant for situation model construction, processes that occur at the phrase and sentence levels (Havas et al., 2007; Simmons et al., 2008).

Indeed, the automaticity of spatial activations for abstract concepts is somewhat suspect. Spatial compatibility effects such as those reported by Meier and Robinson (2004) are not always observed, and their emergence is heavily task dependent (Lebois et al., 2014). For example, Brookshire et al. (2010) presented positively and negatively valenced words in different colored fonts, and asked participants to indicate the font color with button presses that required either an upward or a downward movement. In experimental conditions that encouraged participants to attend to the meaning of the words, upward responses were fastest for positively valenced words, and downward responses were fastest for negatively valenced words. Spatial congruency effects were absent, however, in conditions that encouraged participants to attend only to the color of the words (Brookshire et al., 2010).

Moreover, while the spatial congruency effects reported by, for example, Brookshire et al. (2010) show that word meanings can rapidly influence the motor system, it is unclear whether activity in motor cortex plays any role in the representation of word meanings themselves. Critics of embodied meaning have also argued for the importance of measures with high temporal resolution, noting that fMRI data cannot be used to distinguish between early effects indexing word meaning from later, more strategic simulation effects that might arise from conscious mental imagery (Mahon and Caramazza, 2008). Derived from synaptically generated current flow within patches of neural tissue, event-related brain potentials (ERPs) are a real time measure of brain function that have been associated with numerous aspects of language (Kutas et al., 2006). Here we combined ERP measures of word comprehension with an experimental manipulation intended to modulate activity in the motor system.

Previous ERP studies investigating the action-sentence compatibility (ACE) effect have shown neural responses associated with hand movements that are compatible or incompatible with actions described in sentences (Aravena et al., 2010). When participants judged sentence meaning via button press, they were faster to respond when the shape of their hand was compatible with actions described in the sentences (i.e., open hand press in response to a sentence about clapping). ERPs time locked to sentence-final verbs (e.g., “applauded”) revealed enhanced negativity in a 350–650 ms time window for the incompatible compared to compatible condition. Although the negativity appears to peak at least 600 ms after word onset, the authors describe it as an N400-like effect, and interpret it as an indication that the motor task impacted participants' language comprehension processes.

To further explore sensorimotor contributions to meaning, here we tested whether concurrent hand movements in the vertical plane altered the brain's real time response to words with associated spatial attributes. Accordingly, we recorded participants' EEG as they moved marbles either upwards or downwards while reading words associated with different regions of vertical space. Because spatial associations can be more or less experientially grounded, we compared the impact of our concurrent motion task on words whose verticality was either literal, involving words such as “ascend” and “descend,” or metaphorical, involving words such as “inspire” and “defeat.” The target of the motor act was thus designed to be either congruent or incongruent with the “height” of the word so that we could examine the timing and topographic profile of ERP congruency effects for words whose verticality was either literal or metaphorical.

The use of a single-word reading task allowed us to test predictions of strong embodiment that spatial features are an automatic aspect of meaning activations. If words reactivate associated experiential traces, one would expect the brain's real time response to the words to be modulated by the congruency between participants' hand movements and the vertical features activated by the words. Embodied theories of meaning predict any such congruency effects should arise during the early stages of meaning processing; i.e., within the first 500 ms after word onset. Moreover, embodied theories of metaphor processing suggest broadly similar congruency effects should emerge for words whose verticality is metaphorical, as for words whose verticality is literal.

## Methods

### Participants

This study was conducted with the approval of the UC San Diego Institutional Review Board. Data reported here were from 24 UC San Diego undergraduates (13 male, 11 female). Eleven additional participants were excluded from analysis due to excessive movement artifacts or other technical problems. Participants' ages ranged from 18 to 34, with a mean of 20 years. All participants were right-handed, had normal or corrected-to-normal vision, and none had a history of traumatic head injuries or psychiatric problems. All participants gave informed consent and, in exchange for participation, received extra credit toward their grade in a cognitive science, linguistics, or psychology course.

### Materials

Experimental materials included 84 words in the Literal verticality condition, and 84 words in the Metaphorical verticality condition. Words in the Literal verticality condition were a subset of materials used in Collins (2011), while words in the Metaphorical verticality condition were assembled from materials used in Brookshire et al. (2010) (kindly provided by Daniel Casasanto) and materials published in Meier and Robinson (2004).

Words in the Literal verticality condition were divided into 42 Literal Low words (e.g., descend, floor) and 42 Literal High words (e.g., ascend, ceiling). Words in the Metaphorical verticality condition were divided into 42 Metaphorical Low words (e.g., defeat, poverty) and 42 Metaphorical High words (e.g., inspire, power). See Table 1 for example stimuli. Words in each of the four conditions were roughly matched for psycholinguistic variables as measured by the MRC database (Coltheart, 1981b), including log word frequency (1.9), number of letters (5.4), number of syllables (1.6), familiarity (541.9), and concreteness (433.3). Materials also included 84 “filler” words deemed by experimenters to be relatively neutral with respect to verticality, and similar in log word frequency (1.5), number of letters (5.6), number of syllables (1.7), familiarity (520.8), and concreteness (539.7).

**Table 1:** **Examples of materials from each of the 4 experimental categories**.

**Literal**		**Metaphorical**	
**Low**	**High**	**Low**	**High**
Descend	Ascend	Defeat	Victory
Floor	Ceiling	Poverty	Power
Fall	Leap	Agony	Delight
Puddle	Sky	Theft	Respect

Because Metaphorical Verticality derives from metaphors associating positively valenced items with upper regions of space, and negatively valenced items with lower regions of space, valence and arousal ratings were obtained for these materials from the Affective Norms for English Words (ANEW) 2010 dataset. Valence ratings range from 1 to 9, where 1 is highly negative, 5 is neutral, and 9 is highly positive. Average valence ratings for Metaphorical High words ranged from 6.5 to 8.7 (average = 7.5), whereas ratings for the Metaphorical Low words ranged from 1.5 to 3.28 (average = 2.5). Metaphorical High and Metaphorical Low words were matched on arousal ratings: 5.6 (*SD* = 1.1) vs. 5.7 (*SD* = 0.8), respectively.

The verticality of these materials was established in a separate norming study using 10 new participants drawn from the same pool as the ERP study. Participants in the norming study were instructed to rate each word presented on a 5-point scale from 1 (Very low) to 5 (Very high), and in which 3 signaled “Neither high nor low.” Participants judged both sets of Low words to be below 3, viz. Literal (2.39) and Metaphorical (2.21), and both sets of High words to be above 3, viz. Literal (4.08) and Metaphorical (4.03). The average rating for the filler items was 3.01.

### Procedure and design

Participants were seated in a chair facing a computer monitor located approximately forty inches (101 cm) in front of them. On the floor next to the chair was an apparatus containing approximately 100 black marbles. The apparatus had two wooden trays lined with green or red felt (see Image 1 in Supplementary Materials). The green tray was mounted above the red tray for the entire experiment. Both trays were angled so that the marbles in the trays would roll toward the front, where the participant could easily reach them. Participants moved marbles from one tray to the other while reading words on the computer screen.

Since few ERP studies of language processing involve ongoing, controlled movement, it was unclear whether this paradigm would give rise to lateralized EEG activity reflecting motor control processes unrelated to the movement congruency manipulation. Consequently, half the participants used their left hand throughout the study and half the participants used their right hand. In this way, we hoped to examine the impact of response hand on the ERPs, and whether or not any observed hand effects interacted with experimental variables such as Word direction (low/high), Movement direction (upward/downward), or Lit/Met verticality (literal/metaphorical).

The experiment was divided into four blocks—two in which participants were instructed to move marbles into the red tray, and two in which the green tray was the intended target. Participants changed direction after the second block, and the initial direction (upwards toward the green tray, or downwards toward the red tray) was counterbalanced across participants. At the beginning of each block, participants received verbal instruction indicating which colored tray they should move the marbles into; no language about moving upwards or downwards was used during the instructions. Participants were told their task was to move marbles from one tray to the other while reading words presented on the computer monitor.

At the beginning of each block, participants fixated on the center of the monitor and waited until the word “Ready” appeared. Upon seeing the word “Ready,” they began moving marbles into the specified tray, using only the arm on the ipsilateral side of the tray. The use of the left or right arm was counterbalanced across participants. Participants were asked to move the marbles at a constant rate, without moving their shoulder and without looking at the marble apparatus. Participants were informed that there would be a memory test afterward to ensure they were reading the words. At the end of a block, the experimenter moved the marbles back into the appropriate tray, as necessary.

Each trial was preceded by a small, yellow fixation cross at the center of the screen, followed by the presentation of a word. Words were presented for 500 ms, followed by 1000 ms of fixation cross. Each block involved the presentation of 21 words from each of the four experimental categories (Literal Low, Literal High, Metaphorical Low, and Metaphorical High) as well as 42 fillers. Presentation order was randomized. Each word was presented twice, once accompanied by an upward movement, and once by a downward movement. Words presented in the first block were presented again in the third block in a different random order; likewise the same words were presented in the second and fourth blocks.

Design was thus mixed, with Hand (left/right) as a between-participants variable, and Movement direction (upwards/downwards), Word direction (Low/High), and Lit/Met verticality (literal/metaphorical) as within-participants variables.

### EEG recording and analysis

Participants' EEG was recorded with 29 tin electrodes embedded in an Electro-Cap, and arranged in the International 10–20 configuration. EEG recording was referenced on-line to an electrode placed over the left mastoid, and later re-referenced to an average of activity recorded from left and right mastoids. Blinks were monitored by comparing activity at the FP2 channel with recordings from an electrode under the right eye. Horizontal eye movements were monitored via a bipolar derivation of electrodes placed next to each eye (on the outer canthi). All impedances were kept below 5 kOhms.

Analysis involved mean amplitude measurements taken in four intervals intended to capture ERP components to visually presented words 200–300 ms (P2), 300–500 ms (N400), 500–700 ms (LPC), and 700–1100 ms (slow wave). These time windows were chosen based on the ERP literature on language and memory (reviewed in Kutas and Van Petten, 1994; Kutas et al., 2006), and were similar to those used in previous studies in our laboratory (e.g., Davenport and Coulson, 2011).

**Figure 1:**
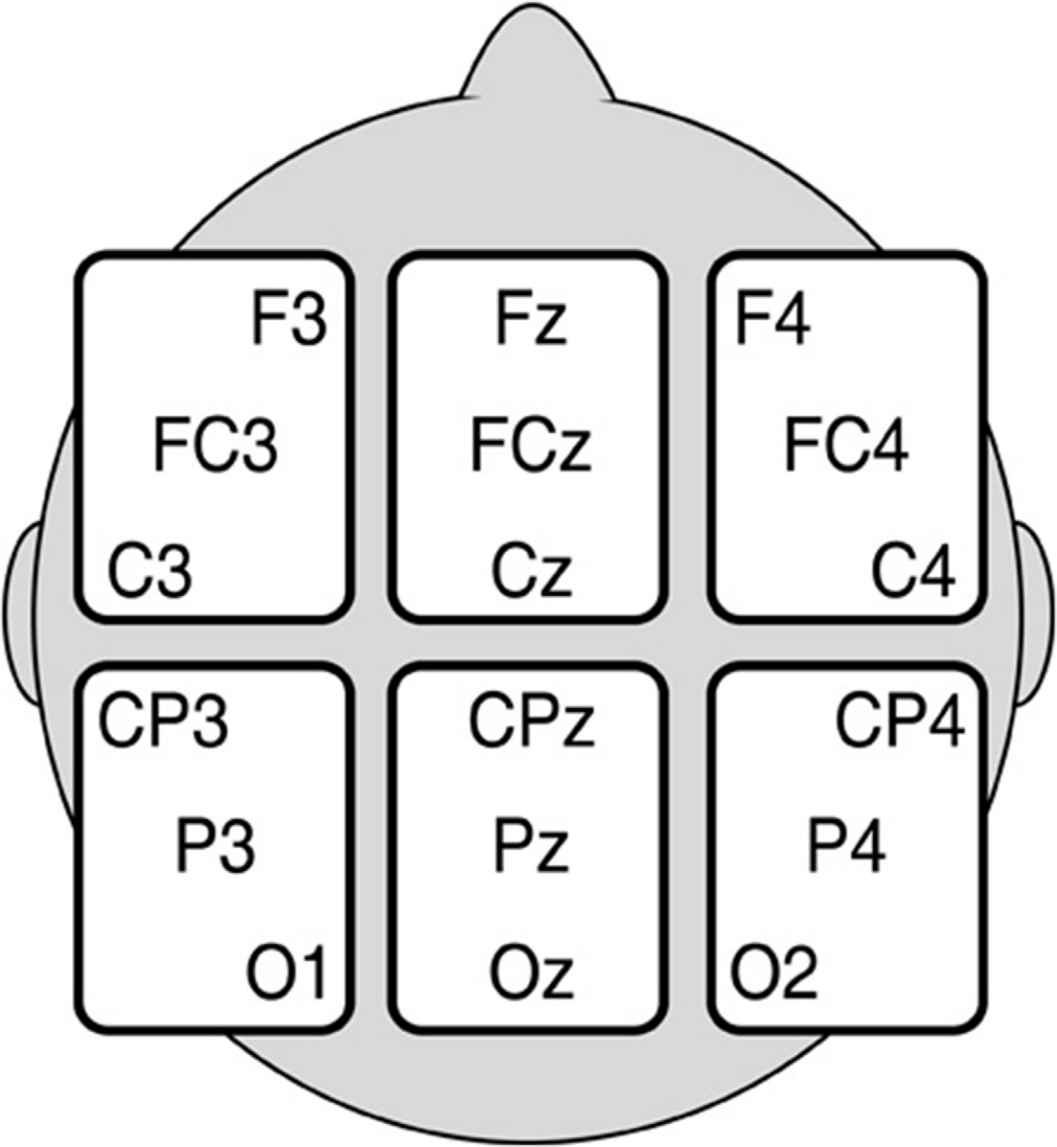
**Electrode sites in each of our regions of interest (ROI)**.

Measurements were subjected to two sets of analyses. The first were a set of pre-planned comparisons motivated by the embodied cognition literature, and were intended to test first, whether literal words would elicit different brain activity in the incongruent than congruent movement condition, and, second, whether metaphorical words would do so. As is customary in these analyses (see Kaschak et al., 2005), Word direction and Movement direction were treated as a single Congruency factor. Accordingly, separate planned comparisons of ERPs to Congruent (low words with downward movements and high words with upward movements) and Incongruent (low words with upward movements and high words with downward movements) stimuli were conducted for Literal and Metaphorical words, respectively. As response hand was counterbalanced, it was not included as a factor in the analysis. Consequently, factors in these planned comparisons included Congruency (congruent/incongruent), Region (6 levels), and Electrode Site (3 levels). The electrode sites included in each Region can be seen in Figure 1.

The second set of analyses were intended to test which, if any, of our independent variables affected the ERPs, and, as such, encompassed all aspects of our design, including the response Hand factor. In these analyses, ERP measurements were subjected to omnibus ANOVA with between-subjects factor Hand (Left/Right), and within-subjects factors Lit/Met verticality (literal, metaphorical), Word direction (low, high), Movement direction (upwards, downwards), Region (6 regions of electrodes) and Electrode site (3 levels). Omnibus analyses enabled us to examine whether there were any unanticipated interactions between experimental variables, and, more importantly, to assess potential differences in congruency effects for literal vs. metaphorical words.

## Results

### ERP measures: planned comparisons

Planned comparisons revealed Congruency effects for words whose verticality was Literal only during the interval 200–300 ms post-onset [Congruency *F*_(1, 23)_ = 5.23, *p* < 0.05; Congruency × Region *F*_(5, 23)_ = 4.05, *p* < 0.05]. Words viewed in the incongruent movement condition elicited slightly more positive ERPs over right frontal electrode sites. Comparable analyses of words whose verticality was metaphorical revealed congruency effects only during the 500–700 ms time window [*F*_(1, 23)_ = 5.57, *p* < 0.05].

### ERP measures: omnibus analyses

#### Early congruency effect

**Figure 2:**
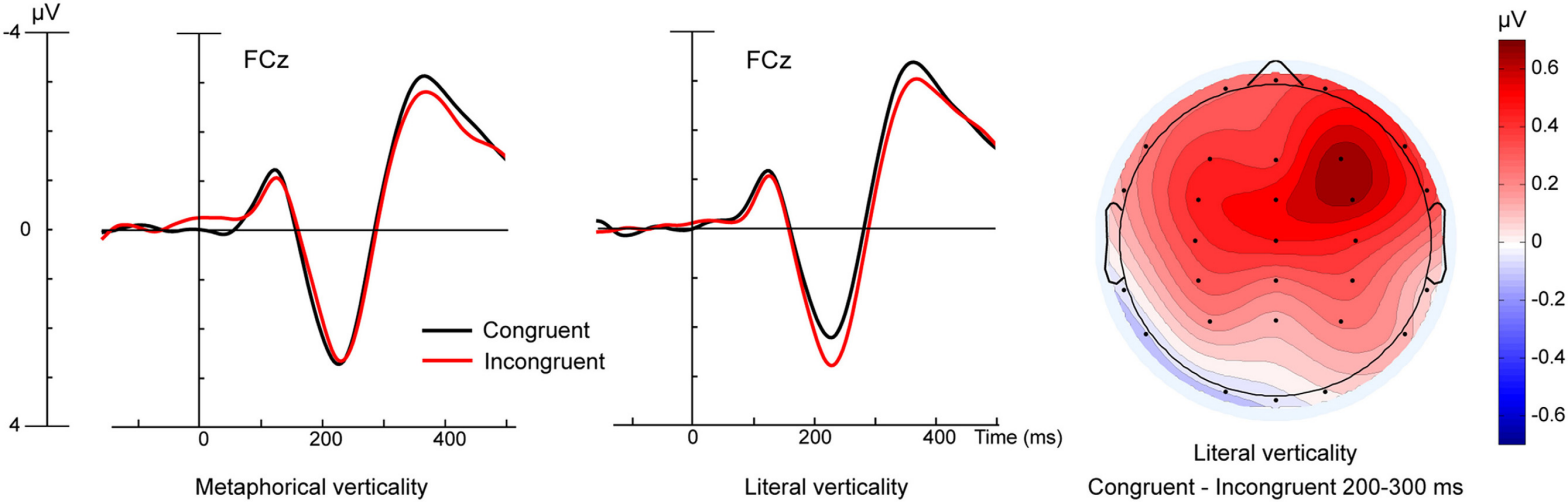
**ERP data at a central electrode for Metaphorical (left) and Literal (right) verticality stimuli**. ERP traces are shown for Congruent (e.g., High words viewed during Upward movement) and Incongruent (e.g., High words viewed during Downward movement) conditions. The rightmost panel depicts a topographic scalp map of the mean amplitude voltage difference for the Literal verticality condition in the 200–300 ms time window.

Analysis of ERPs measured 200–300 ms post-word onset revealed a significant four-way interaction of Lit/Met x Word direction x Movement direction x Electrode [*F*_(5, 110)_ = 3.21, *p* < 0.05]. Results of the planned comparisons suggest this interaction reflects the presence of congruency effects in the literal words coupled with their absence in the metaphorical words. Early congruency effects for literal but not metaphorical words can be seen in Figure 2.

#### Valence effect

Analysis of ERPs measured 300–500 ms post-onset revealed an interaction of Word direction x Lit/Met [*F*_(1, 22)_ = 5.38, *p* < 0.05]. This interaction was followed up with separate repeated measures ANOVAs for literal and metaphorical words. Analysis of words whose verticality was literal revealed no experimental effects.

**Figure 3:**
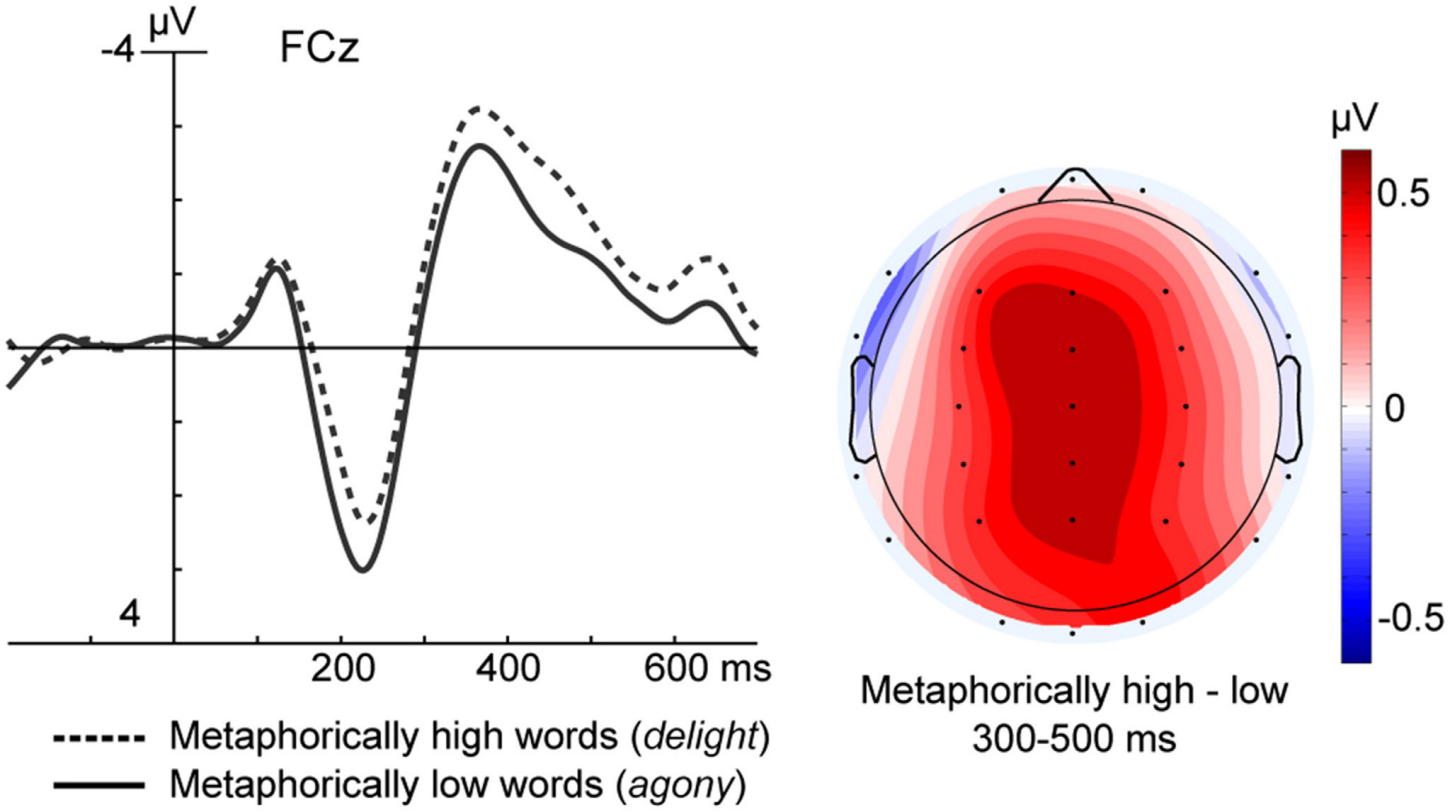
**The left panel shows ERP data at a fronto-central electrode for the Metaphorically high (i.e., positively valenced) and Metaphorically low (i.e., negatively valenced) conditions collapsed across movement direction**. The right panel depicts a topographic scalp map of the mean amplitude voltage difference for the Metaphorical verticality conditions in the 300–500 ms time window.

By contrast, analysis of words whose verticality was metaphorical revealed a main effect of Word direction [*F*_(1, 22)_ = 6.64, *p* < 0.05]. Relative to metaphorical low words, metaphorical high words elicited slightly less negative ERPs over central scalp sites (see Figure 3). As these differences a) did not interact with movement direction and b) were not observed for the affectively neutral items (viz. the literal words, or, for that matter, the filler words), Word direction effects in the metaphorical words were presumed to be due to the fact that high words were positively valenced, while the low words were negatively valenced^1^.

#### Late congruency effects

**Figure 4:**
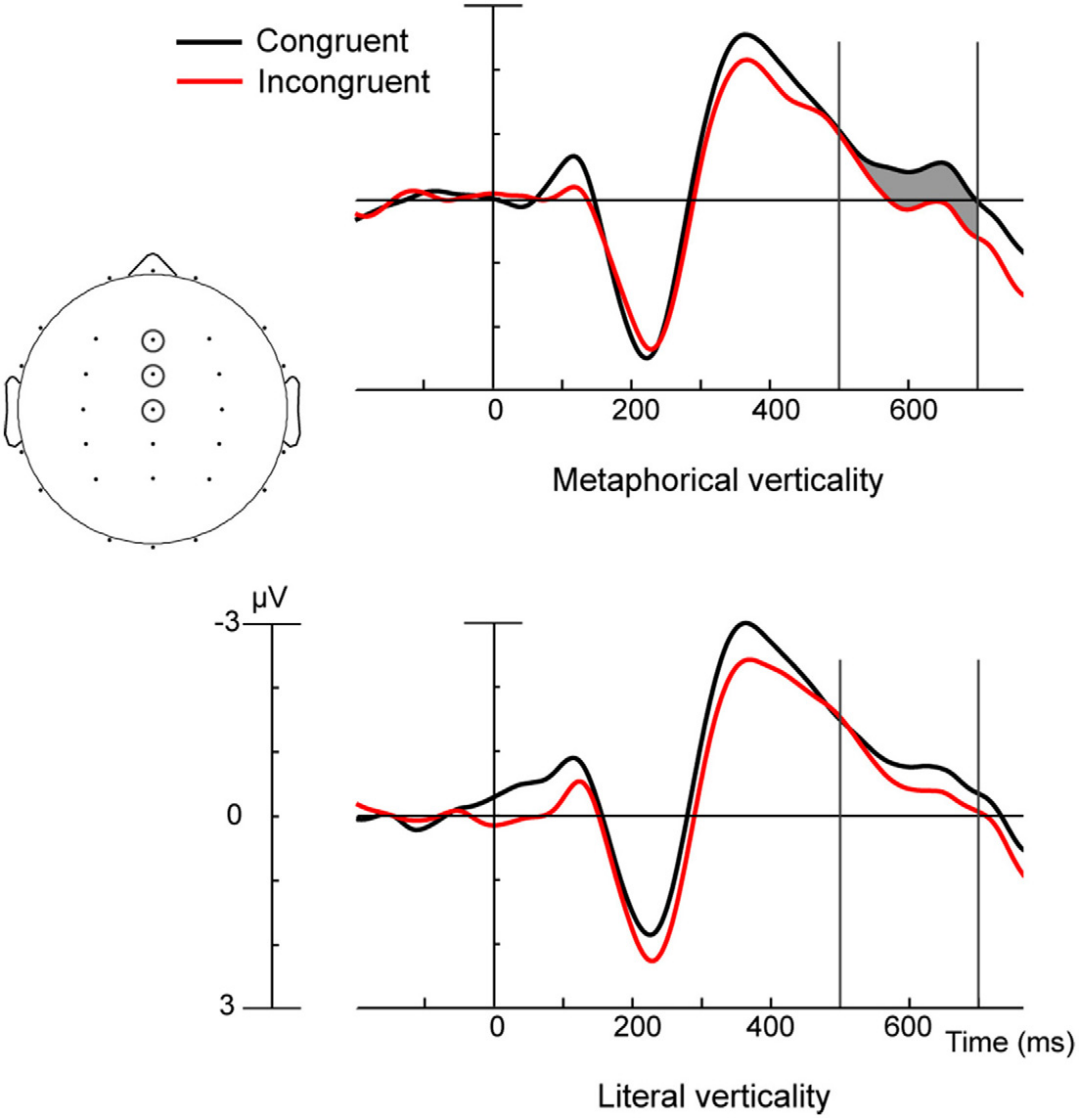
**ERPs to Congruent (black) and Incongruent (red) words in the Metaphorical condition (top panel) and in the Literal condition (bottom panel)**. ERPs shown reflect a composite of the fronto-central ROI electrodes circled and were taken from participants using their left hand. Shaded area represents significant congruency effects 500–700 ms for Metaphorical condition only.

Analysis of ERPs measured 500–700 ms post-word onset revealed a Hand x Region x Electrode site interaction [*F*_(10, 220)_ = 4.64, *p* < 0.01], as participants using their Left hand had more positive ERPs over frontal central electrodes than those using their Right hand. As this contrasted with the Congruency effect observed in our planned comparisons of ERPs time locked to metaphors and which collapsed across the Hand factor (see Section ERP Measures: Planned Comparisons), we conducted separate *post-hoc* analyses of ERPs recorded from participants using their Left hand, and those using their Right hand. In each group, ERPs to metaphors were measured 500–700 ms and subjected to repeated measures ANOVA with factors Congruency (congruent/incongruent), Region (6 levels), and Electrode (3 levels). A significant congruency effect was found for participants using their Left hand [*F*_(1, 11)_ = 4.86, *p* < 0.05], but not for participants using their Right hand [*F*_(1, 11)_ = 1.53, *p* = 0.24]. The discrepancy between the effect of Hand in the omnibus analysis and the effect of Congruency in the planned comparisons presumably results because the Metaphor Congruency effect is driven by ERPs in the group who moved the marbles with their non-dominant hand. As one might expect from the planned comparisons, analyses of the ERPs to words whose verticality was literal showed no sign of Congruency effects in either the group using their Left hand LH [*F*_(1, 11)_ = 1.22, *p* = 0.29] or their Right hand [*F*_(1, 11)_ = 1.13, *p* = 0.31]. ERPs from the group using their Left hand are shown in Figure 4. Reliable Congruency effects were observed 500–700 ms after the onset of Metaphorical but not Literal words.

**Figure 5:**
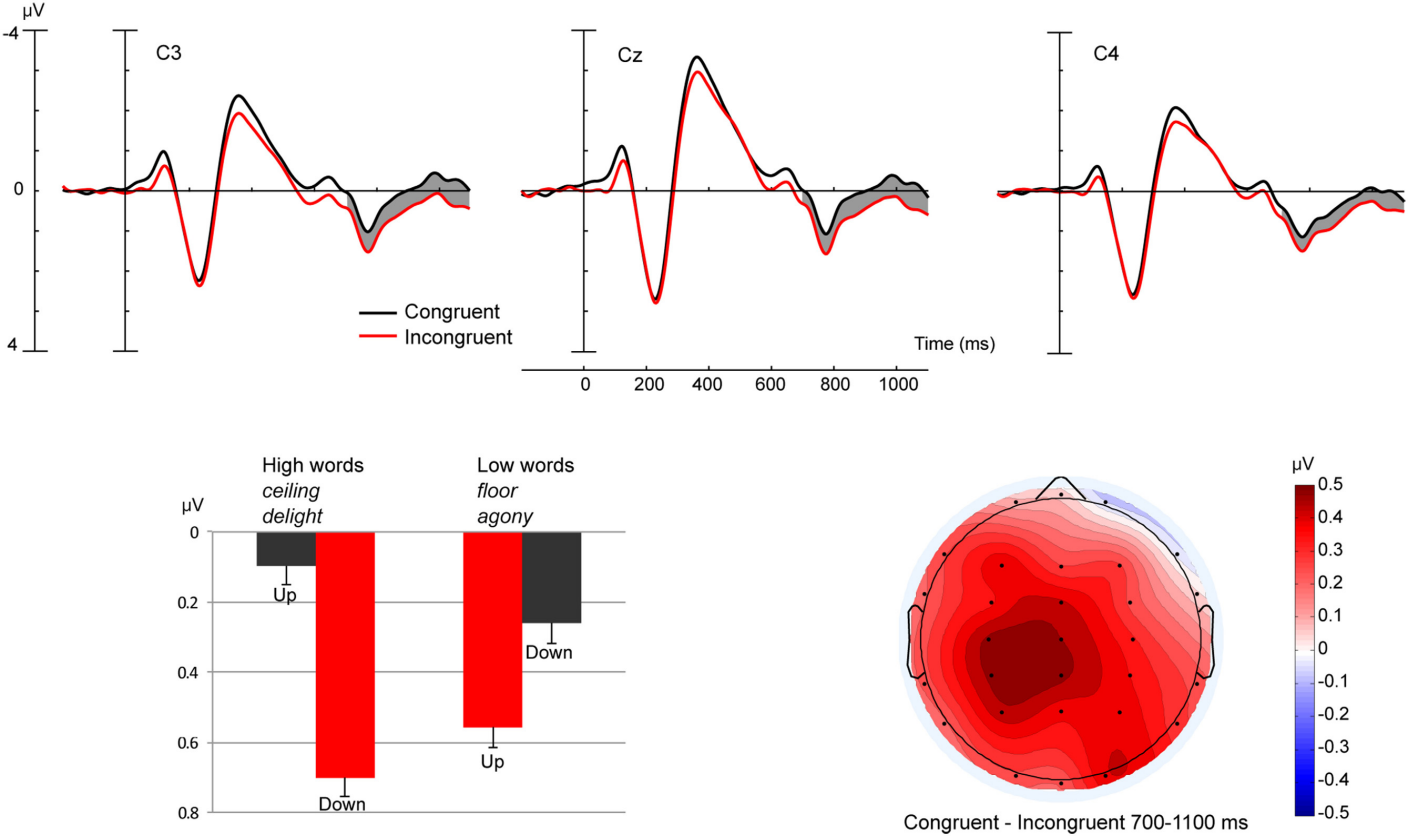
**The top three panels show ERP data at three central electrodes for participants using their left hand**. ERP traces are shown for Congruent and Incongruent conditions collapsed across Lit/Met. The bottom left panel shows a bar graph of ERP mean amplitudes averaged across all electrodes in the 700–1100 ms time window. Error bars indicate SEM. The bottom right panel depicts a topographic scalp map of the mean amplitude voltage difference for the collapsed Lit/Met conditions for participants using their left hand.

Omnibus analysis of the 700–1100 ms time window revealed an interaction between Hand and Lit/Met [*F*_(1, 22)_ = 4.92, *p* < 0.05] and Hand, Word Direction, and Movement Direction [*F*_(1, 22)_ = 6.69, *p* < 0.05]. To further explore these interactions with Hand group, we conducted two *post-hoc* ANOVAs—one for each Hand group—each with factors Lit/Met (literal/metaphorical), Word Direction (high/low), Movement Direction (upwards/downwards), Region (6 levels), and Electrode site (3 levels). Analysis of participants using their Right Hand did not reveal any experimental effects. For participants using their Left Hand, analysis revealed a Word direction x Movement direction interaction [*F*_(1, 11)_ = 7.30, *p* < 0.05], i.e., a Congruency effect. Figure 5 shows that ERPs were more positive for words in the incongruent movement condition. As no interactions with the Lit/Met factor were observed, these effects were presumed to be similar for literal and metaphorical words. Moreover, analysis of ERPs recorded from participants using their Left Hand indicated Congruency effects just missed significance in both Literal [*F*_(1, 11)_ = 4.66, *p* = 0.054] and Metaphorical [*F*_(1, 11)_ = 4.40, *p* = 0.06] words.

### Memory test

Due to time constraints on the experimental session (viz. all experiments had to be completed within a 2-h time span), only 22 of our 24 participants completed the memory test administered at the conclusion of the marbles task. The memory test for each participant was comprised of a random subset of “old” words, along with an equivalent number of “new” words that had not been presented during the experiment. Participants correctly recognized an average of 40% of the “old” words, with an average false alarm rate of 16.1%. Repeated measures ANVOA with the between-subjects factor Hand, and within-subjects factors Lit/Met, and Word Direction revealed only a main effect of Lit/Met [*F*_(1, 20)_ = 7.1, *p* < 0.05], with higher hit rates for Metaphorical (45%) than Literal (36.4%) words.

## Discussion

To explore the contributions of motor cortex to the processing of literal and metaphorical language, the present study examined the event-related brain response to visually presented words as participants performed a concurrent movement task. In keeping with embodied theories of meaning, we found that words whose verticality was literal (e.g., ceiling, floor) elicited more positive ERPs 200–300 ms post-word onset when accompanied by incongruent movements than congruent ones. Effects of movement congruency did not impact ERPs to words whose verticality was metaphorical until more than 500 ms after word onset. During the late interval 700–1100 ms, both literal and metaphorical words elicited more positive ERPs in the incongruent movement condition. Observed effects argue against a strong view of embodied metaphor that predicts early, bottom-up sensorimotor contributions to metaphoric meanings. However, the late congruency effects for the metaphorical items suggests participants were sensitive to a connection between abstract concepts and particular regions of vertical space in accordance with the orientation metaphor GOOD IS UP.

### Early congruency effects for literal words

Early congruency effects observed for the literal condition are consistent with work in cognitive psychology suggesting words depicting concrete objects and actions can activate spatial features with some modal content. For example, Zwaan and Yaxley (2003) showed that participants were faster to judge that pairs of words, such as “attic” and “basement,” were related to one another when they were arranged iconically with “attic” above “basement” than for the reverse configuration. Behavioral research also supports interaction between language and the visual system, as words associated with either high (“hat”) or low (“boot”) regions of space modulate visual attention in those regions (Estes et al., 2008). Moreover, responses to words such as “head” and “foot” have been shown to be faster when they involve a movement that is congruent with the words' spatial associations than incongruent, suggesting that words can evoke spatial schemas with a motor component (Borghi et al., 2004).

Not only do results of the present study support behavioral demonstrations of cross-talk between language and overt motor behavior, they go beyond those findings by showing the impact of the movement manipulation on the real time processing of the words themselves. We suggest that the congruency effect shown in Figure 2 reflects greater activity in motor or premotor cortex due to the incompatibility of the vertical hand movements with the vertical features evoked by the words. In support of this interpretation, the 200 ms onset of the congruency effect is coincident with the time at which semantic processing of action verbs begins to influence movement kinematics (Boulenger et al., 2006). The interval 200 ms post-onset was also the interval in which Hauk and Pulvermüller (2004) observed a somatotopic response profile in ERPs to action verbs such as “kick” and “lick,” consistent with a generator in motor and premotor cortex (Shtyrov et al., 2004).

The timing and topography of the observed congruency effect was also reminiscent of differences in the electrophysiological response to action verbs associated with motor features, and concrete nouns associated with a preponderance of visual features. Relative to nouns, action verbs elicit enhanced positivities over fronto-central electrodes beginning 200 ms post-onset, argued to reflect greater activity in motor and premotor cortices (Preissl et al., 1995; Pulvermüller et al., 1999a). Similar effects have been observed in the electrophysiological response to nouns with predominantly motor vs. predominantly visual associations, indicating these positivities do not stem from syntactic differences between nouns and verbs (Pulvermüller et al., 1999b).

The results of the current study add to the findings reviewed above by showing that engaging the motor system during a reading task can change the early ERP response depending on whether the task supports or hinders embodied processing of a single word. In sum, the early congruency effect for literal words may reflect increased recruitment of motor and premotor cortex engendered by the conflicting demands of the movement task and the motoric features activated by the words. As such, data from the present study provide support for the embodiment claim that concrete concepts have a perceptuo-motor basis and recruit brain structures involved in perception and action.

### N400 interval

One perhaps surprising finding in the present study was that the experimental manipulation, that is, directing participants to move marbles either upwards or downwards, had no detectable effects on the portion of the ERP waveform most consistently linked to semantic processing: the N400 component. These data contrast with prior reports of N400 effects resulting from experimental manipulations that varied the availability of modal information (Kellenbach et al., 2000; Collins et al., 2011; Hald et al., 2011). The present study differed from previous work, however, in its focus on motoric aspects of word meaning, as opposed to perceptual ones, such as the auditory or visual features of objects (Chwilla et al., 2007; Collins et al., 2011). Moreover, research on the neural substrate of action verb processing suggests motoric features are activated 200–300 ms after word onset (Pulvermüller, 2005), and perhaps precede the activation of perceptual features that have been shown to influence the amplitude of the N400 component (Chwilla et al., 2007).

Although it is unwise to read too much into a null result, the absence of N400 congruency effects in the present study are a poor fit with strong embodied meaning models that suggest words elicit fast, automatic sensorimotor activations that operate similarly across contexts. Moreover, the absence of N400 congruency effects in the present study contrasts with the report that verbs such as “applauded” elicit greater N400 when the experimental task requires an incongruent response (involving a clenched fist) than a congruent one (involving an open hand) (Aravena et al., 2010; Ibáñez et al., 2013). One crucial difference between these ERP studies of the action sentence compatibility effect and the present study is the use of single words rather than sentences. The disparity between our findings here with those reported by Aravena et al. (2010) is easily reconciled by models of embodied meaning that suggest sensorimotor activations result from sentence- rather than word- level meaning construction (Zwaan, 2004). Different from strong embodiment models, these models suggest that words prompt listeners to construct sensorimotor simulations that unfold over time. These weak embodiment models posit contextual variability in conceptual activations associated with words, and depict simulation as more of a strategic process (e.g., Lebois et al., 2014).

Supporters of a weak embodiment approach, then, might explain our failure to observe movement congruency effects by noting that the present study did not promote deep enough processing to impact the semantic retrieval operations indexed by the N400. Our task was simply to read the words while performing the movement task, and while participants were told that there would be a memory task, it was not administered until the very end of the ERP recording session. Behavioral research on spatial schemas evoked by words such as “sky” and “ground” indicates the emergence of spatial congruency effects depends on tasks that highlight these words' spatial features (Brookshire et al., 2010; Lebois et al., 2014). Future work should examine how tasks designed to increase the depth of processing impact early vs. late ERP congruency effects observed in the present study.

Another explanation of the absence of congruency effects 300–500 ms post-onset is that the design lacked adequate power. We find this unlikely, however, because we did observe reliable differences during this interval between metaphorically high words such as “delight” and metaphorically low words such as “agony” (see Figure 3). Given that no such difference was observed for literal high and low words that were matched in affective valence, we attribute this effect to the fact that the high words were positively valenced, while the low words were negative. The timing and topography of observed valence effects were in keeping with previous studies of affectively valenced words (see Fischler and Bradley, 2006 for a review), and did not interact with the movement condition.

### Late congruency effects

In contrast to the literally related words, metaphorically related words did not elicit movement congruency effects until after 500 ms, arguing against a strong embodied metaphor theory. Instead, metaphorical congruency effects emerged later, with incongruent words eliciting more positive ERPs than congruent ones in the latter part of the epoch. Congruency effects suggest that participants were sensitive to the purely metaphorical verticality of words such as “inspire” and “defeat.” However, the lateness of these effects suggests they index processing that follows the initial access of meaning. Indeed, congruency effects for the metaphors presumably reflect the same sorts of intuitions that the participants in our norming study employed to assign a verticality rating to words such as “delight.”

Interestingly, single words do not usually elicit late positive effects unless they are employed as a part of a judgment task. Late positivities are more commonly elicited by words in sentences and larger discourses (Van Petten and Luka, 2012), and the amplitude of the late positive complex has been argued to reflect the cost of integrating a word into the larger context (Brouwer et al., 2012). In the present study, the ongoing movement task presumably served as the context, and larger positivities to the incongruent words may reflect the need for additional processing when the direction of planned motion does not match the verticality of the presented word. Consistent with this interpretation, the amplitude of the late positivity is enhanced by response conflict (Doucet and Stelmack, 1999), especially in spatial compatibility paradigms (Leuthold and Sommer, 1999).

Late emerging congruency effects might thus be argued to support a weak form of embodied metaphor in which sensorimotor simulations arise in late stages of meaning processing, perhaps to guide pragmatic inferences. In keeping with this suggestion, late positivities in ERPs to linguistic stimuli have been associated with a number of pragmatic phenomena that require inferential operations, such as the comprehension of jokes (Coulson and Lovett, 2004), ironic remarks (Regel et al., 2010), metaphors (Coulson and Van Petten, 2002), and semantic novelty (Davenport and Coulson, 2011). Indeed, spatial schemas are often used to evoke pragmatic inferences, as in a performance evaluation that reads, “This employee has reached rock bottom, yet continues to dig.”

Somewhat different than strong embodied models such as that suggested by Gallese and Lakoff (2005), we suggest a more tempered version of embodied metaphor in which the deployment of sensorimotor simulations is not automatic, but, rather, depends on strategic factors. Indeed, strong embodiment might be understood as adopting an overly reflexive model of lexical activation in which a given word gives rise to a definitive sensorimotor simulation. Many psycholinguists have eschewed the early idea that words automatically activate a fixed lexical entry, suggesting instead that they prompt context-sensitive retrieval from semantic memory (e.g., Coulson, 2006; Elman, 2009). Likewise, some advocates of grounded cognition have suggested that the linguistic activation of modal information varies as a function of context and task (Louwerse and Jeuniaux, 2008; Lebois et al., 2014).

On such a view, the relevance of applicable conceptual metaphors varies as a function of context in much the same way that the relevance of other conceptual information does. As a result, the mere reading of a word such as “wealth” will not necessarily elicit the spatial activations derived from the metaphor GOOD IS UP. Rather, contextual factors will render those associations more or less relevant. In the present study, the cognitive set induced by the movement task may have enhanced the salience of the orientation metaphor, making participants more sensitive to the congruency between concepts such as wealth and upwards-directed movements.

### Summary

The early congruency effect for literal words may reflect increased recruitment of motor and premotor cortex engendered by the conflicting demands of the movement task and the motoric features activated by the words. As such, data from the present study provide support for the embodiment claim that concrete concepts have a perceptuo-motor basis and recruit brain structures involved in perception and action. By similar reasoning, the absence of comparable congruency effects for the metaphors argues against embodied metaphor theories that posit rapid, bottom-up activation of sensorimotor cortex as part of word comprehension. Late emerging congruency effects for metaphors are more consistent with weak embodiment that posits strategic connections between abstract concepts and spatial schemas. Whereas the early congruency effects reflect the impact of the movement task on processing word meaning, the late congruency effects may reflect the availability of spatial schemas for pragmatic inference.

### Conflict of interest statement

The authors declare that the research was conducted in the absence of any commercial or financial relationships that could be construed as a potential conflict of interest.

## Footnotes

^1^Based on the suggestion of a reviewer, we analyzed ERPs for metaphorical high and low words separately to investigate whether either type of word (positive or negative valence) yielded a congruency effect of Word direction x Motion direction. There was a trend toward a significant Word direction x Motion direction interaction for metaphorical high words [*F*_(1, 23)_ = 3.49, *p* = 0.07], with high words viewed in the downward condition (incongruent) eliciting more positive ERPs than in the upward condition (congruent) (μ = 2.13, μ = 1.67 μV). However, given the unmotivated nature of these analyses, it would be unwise to draw any conclusions from this non-significant trend.

## References

[B1] Aravena P. Hurtado E. Riveros R. Cardona J. F. Manes F. Ibáñez A. (2010). Applauding with closed hands: neural signature of action-sentence compatibility effects. PLoS ONE 5:e11751. 10.1371/journal.pone.0011751 20676367 PMC2911376

[B2] Barsalou L. W. (2008). Grounded cognition. Annu. Rev. Psychol. 59, 617–645. 10.1146/annurev.psych.59.103006.093639 17705682

[B3] Barsalou L. W. (2010). Grounded cognition: past, present, and future. Top. Cogn. Sci. 2, 716–724. 10.1111/j.1756-8765.2010.01115.x 25164052

[B3a] Borghi A. M. Glenberg A. M. Kaschak M. P. (2004). Putting words in perspective. Mem. Cognit. 32, 863–873. 10.3758/BF03196865 15673175

[B4] Boroditsky L. (2011). How languages construct time, in Space, Time and Number in the Brain: Searching for the Foundations of Mathematical Thought, eds Dehaene S. Brannon E. (London: Elsevier), 333–341.

[B5] Boulenger V. Roy A. C. Paulignan Y. Deprez V. Jeannerod M. Nazir T. A. (2006). Cross-talk between language processes and overt motor behavior in the first 200 msec of processing. J. Cogn. Neurosci. 18, 1607–1615. 10.1162/jocn.2006.18.10.1607 17014366

[B6] Brookshire G. Ivry R. Casasanto D. (2010). Modulation of motor-meaning congruency effects for valenced words, in Proceedings of the 32nd Annual Conference of the Cognitive Science Society (Austin, TX), 1940–1945.

[B7] Brouwer H. Fitz H. Hoeks J. (2012). Getting real about semantic illusions: rethinking the functional role of the P600 in language comprehension. Brain Res. 1446, 127–143. 10.1016/j.brainres.2012.01.055 22361114

[B8] Chwilla D. J. Kolk H. H. Vissers C. T. (2007). Immediate integration of novel meanings: N400 support for an embodied view of language comprehension. Brain Res. 1183, 109–123. 10.1016/j.brainres.2007.09.014 17950260

[B9a] Collins J. B. (2011). The Integration of Language with Perceptual Systems. Ph.D. dissertation, University of California, Sandiego

[B9] Collins J. Pecher D. Zeelenberg R. Coulson S. (2011). Modality switching in a property verification task: an ERP study of what happens when candles flicker after high heels click. Front. Psychol. 2:10. 10.3389/fpsyg.2011.00010 21713128 PMC3111443

[B10] Coltheart M. (1981b). The MRC psycholinguistic database. Q. J. Exp. Psychol. 33A, 497–505 10.1080/14640748108400805

[B11] Coulson S. (2006). Constructing meaning. Metaphor. Symbol 21, 245–266 10.1207/s15327868ms2104_3

[B12] Coulson S. Lovett C. (2004). Handedness, hemispheric asymmetries, and joke comprehension. Cogn. Brain Res. 19, 275–288. 10.1016/j.cogbrainres.2003.11.015 15062865

[B13] Coulson S. Van Petten C. (2002). Conceptual integration and metaphor: an event-related potential study. Mem. Cogn. 30, 958–968. 10.3758/BF03195780 12450098

[B14] Davenport T. Coulson S. (2011). Predictability and novelty in literal language comprehension: an ERP study. Brain Res. 1418, 70–82. 10.1016/j.brainres.2011.07.039 21925647

[B15] Doucet C. Stelmack R. M. (1999). The effect of response execution on P3 latency, reaction time, and movement time. Psychophysiology 36, 351–363. 10.1017/S0048577299980563 10352559

[B16] Elman J. L. (2009). On the meaning of words and dinosaur bones: lexical knowledge without a lexicon. Cogn. Sci. 33, 547–582. 10.1111/j.1551-6709.2009.01023.x 19662108 PMC2721468

[B17] Estes Z. Verges M. Barsalou L. W. (2008). Head up, foot down object words orient attention to the objects' typical location. Psychol. Sci. 19, 93–97. 10.1111/j.1467-9280.2008.02051.x 18271853

[B18] Fischer M. H. Zwaan R. A. (2008). Embodied language: a review of the role of the motor system in language comprehension. Q. J. Exp. Psychol. 61, 825–850. 10.1080/17470210701623605 18470815

[B19] Fischler I. Bradley M. (2006). Event-related potential studies of language and emotion: words, phrases, and task effects. Prog. Brain Res. 156, 185–203. 10.1016/S0079-6123(06)56009-1 17015080

[B20] Gallese V. Lakoff G. (2005). The brain's concepts: the role of the sensory-motor system in conceptual knowledge. Cogn. Neuropsychol. 22, 455–479. 10.1080/02643290442000310 21038261

[B21] Glenberg A. M. Kaschak M. P. (2002). Grounding language in action. Psychon. Bull. Rev. 9, 558–565. 10.3758/BF03196313 12412897

[B22] Hald L. A. Marshall J. A. Janssen D. P. Garnham A. (2011). Switching modalities in a sentence verification task: ERP evidence for embodied language processing. Front. Psychol. 2:45. 10.3389/fpsyg.2011.00045 21779254 PMC3132671

[B23] Hauk O. Pulvermüller F. (2004). Neurophysiological distinction of action words in the fronto−central cortex. Hum. Brain Mapp. 21, 191–201. 10.1002/hbm.10157 14755838 PMC6872027

[B23a] Havas D. A. Glenberg A. M. Rinck M. (2007). Emotion simulation during language comprehension. Psychon. Bull. Rev. 14, 436–441. 10.3758/BF03194085 17874584

[B24] Head H. (1926). Aphasia and Kindred disorders of speech, Vol. 1 London: Cambridge University Press.

[B25] Ibáñez A. Cardona J. F. Dos Santos Y. V. Blenkmann A. Aravena P. Roca M.. (2013). Motor-language coupling: direct evidence from early Parkinson's disease and intracranial cortical recordings. Cortex 49, 968–984. 10.1016/j.cortex.2012.02.014 22482695

[B26] Kaschak M. P. Madden C. J. Therriault D. J. Yaxley R. H. Aveyard M. Blanchard A. A.. (2005). Perception of motion affects language processing. Cognition 94, B79–B89. 10.1016/j.cognition.2004.06.005 15617669

[B27] Kellenbach M. L. Wijers A. A. Mulder G. (2000). Visual semantic features are activated during the processing of concrete words: event-related potential evidence for perceptual semantic priming. Cogn. Brain Res. 10, 67–75. 10.1016/S0926-6410(00)00023-9 10978693

[B28] Kovecses Z. (2006). Language, Mind, and Culture: A Practical Introduction. New York, NY: Oxford.

[B29] Kutas M. Van Petten C. (1994). Psycholinguistics electrified, in Handbook of Psycholinguistics, ed Gernsbacher M. A. (Academic Press), 83–143.

[B30] Kutas M. Van Petten C. Kluender R. (2006). Psycholinguistics electrified II: 1994-2005, in Handbook of Psycholinguistics, 2nd Edn., eds Traxler M. Gernsbacher M. A. (New York, NY: Elsevier), 659–724.

[B31] Lakoff G. Johnson M. (1980). Metaphors we Live by. Chicago: University of Chicago Press.

[B32] Lakoff G. Johnson M. (1999). Philosophy in the Flesh: The Embodied Mind and Its Challenge to Western Thought. New York, NY: Basic books.

[B33] Lebois L. A. Wilson-Mendenhall C. D. Barsalou L. W. (2014). Are automatic conceptual cores the gold standard of semantic processing? The context-dependence of spatial meaning in grounded congruency effects. Cogn. Sci. [Epub ahead of print]. 10.1111/cogs.12174 25243925

[B34] Leuthold H. Sommer W. (1999). ERP correlates of error processing in spatial SR compatibility tasks. Clin. Neurophysiol. 110, 342–357. 10.1016/S1388-2457(98)00058-3 10210624

[B35] Louwerse M. M. Jeuniaux P. (2008). Language comprehension is both embodied and symbolic, in Symbols and Embodiment: Debates on Meaning and Cognition, eds Glenberg A. M. Graesser A. C. de Vega M. (Oxford: Oxford University Press), 309–326.

[B36] Mahon B. Z. Caramazza A. (2008). A critical look at the embodied cognition hypothesis and a new proposal for grounding conceptual content. J. Physiol. Paris 102, 59–70. 10.1016/j.jphysparis.2008.03.004 18448316

[B37] Meier B. P. Robinson M. D. (2004). Why the sunny side is up associations between affect and vertical position. Psychol. Sci. 15, 243–247. 10.1111/j.0956-7976.2004.00659.x 15043641

[B38] Meteyard L. Cuadrado S. R. Bahrami B. Vigliocco G. (2012). Coming of age: a review of embodiment and the neuroscience of semantics. Cortex 48, 788–804. 10.1016/j.cortex.2010.11.002 21163473

[B39] Preissl H. Pulvermüller F. Lutzenberger W. Birbaumer N. (1995). Evoked potentials distinguish between nouns and verbs. Neurosci. Lett. 197, 81–83. 10.1016/0304-3940(95)11892-Z 8545063

[B40] Pulvermüller F. (2005). Brain mechanisms linking language and action. Nat. Rev. Neurosci. 6, 576–582. 10.1038/nrn1706 15959465

[B41] Pulvermüller F. Lutzenberger W. Preissl H. (1999a). Nouns and verbs in the intact brain: evidence from event-related potentials and high-frequency cortical responses. Cereb. Cortex 9, 497–506. 10.1093/cercor/9.5.497 10450894

[B42] Pulvermüller F. Mohr B. Schleichert H. (1999b). Semantic or lexico-syntactic factors: what determines word-class specific activity in the human brain? Neurosci. Lett. 275, 81–84. 10.1016/S0304-3940(99)00724-7 10568504

[B43] Pylyshyn Z. W. (1984). Computation and Cognition. Cambridge, MA: MIT Press.

[B44] Regel S. Coulson S. Gunter T. C. (2010). The communicative style of a speaker can affect language comprehension? ERP evidence from the comprehension of irony. Brain Res. 1311, 121–135. 10.1016/j.brainres.2009.10.077 19900421

[B45] Santana E. De Vega M. (2011). Metaphors are embodied, and so are their literal counterparts. Front. Psychol. 10:90. 10.3389/fpsyg.2011.00090 21687459 PMC3110336

[B46] Schubert T. W. (2005). Your highness: vertical positions as perceptual symbols of power. J. Pers. Soc. Psychol. 89:1. 10.1037/0022-3514.89.1.1 16060739

[B47] Shtyrov Y. Hauk O. Pulvermüller F. (2004). Distributed neuronal networks for encoding category−specific semantic information: the mismatch negativity to action words. Eur. J. Neurosci. 19, 1083–1092. 10.1111/j.0953-816X.2004.03126.x 15009156

[B48] Simmons W. K. Hamann S. B. Harenski C. L. Hu X. P. Barsalou L. W. (2008). fMRI evidence for word association and situated simulation in conceptual processing. J. Physiol. Paris 102, 106–119. 10.1016/j.jphysparis.2008.03.014 18468869

[B49] Van Petten C. Luka B. J. (2012). Prediction during language comprehension: benefits, costs, and ERP components. Int. J. Psychophysiol. 83, 176–190. 10.1016/j.ijpsycho.2011.09.015 22019481

[B50] Wernicke C. (1874/1977). Der aphasische symptomencomplex: eine psychologische studie auf anatomischer basis, in Wernicke's Works on Aphasia: a Sourcebook and Review, ed Eggert G. H. (The Hague: Mouton), 91–145.

[B51] Zwaan R. A. (2004). The immersed experiencer: toward an embodied theory of language comprehension. Psychol. Learn. Motiv. 44, 35–62 10.1016/S0079-7421(03)44002-4

[B52] Zwaan R. A. Madden C. J. (2005). Embodied sentence comprehension, in The Grounding of Cognition: The Role of Perception and Action in Memory, Language, and Thinking, eds Pecher D. Zwaan R. A. (Cambridge: Cambridge University Press), 224–245.

[B53] Zwaan R. A. Yaxley R. H. (2003). Spatial iconicity affects semantic relatedness judgments. Psychon. Bull. Rev. 10, 954–958. 10.3758/BF03196557 15000544

