# Supplementary material for: How vertical hand movements impact brain activity elicited by literally and metaphorically related words: an ERP study of embodied metaphor
Source: Front Hum Neurosci. 2014 Dec 23;8:1031. doi: 10.3389/fnhum.2014.01031 (PMC4274969; doi:10.3389/fnhum.2014.01031)

Figure 1. Cartoon of experimental setup showing participant, green and red marble trays, computer screen, and EEG amplifier.

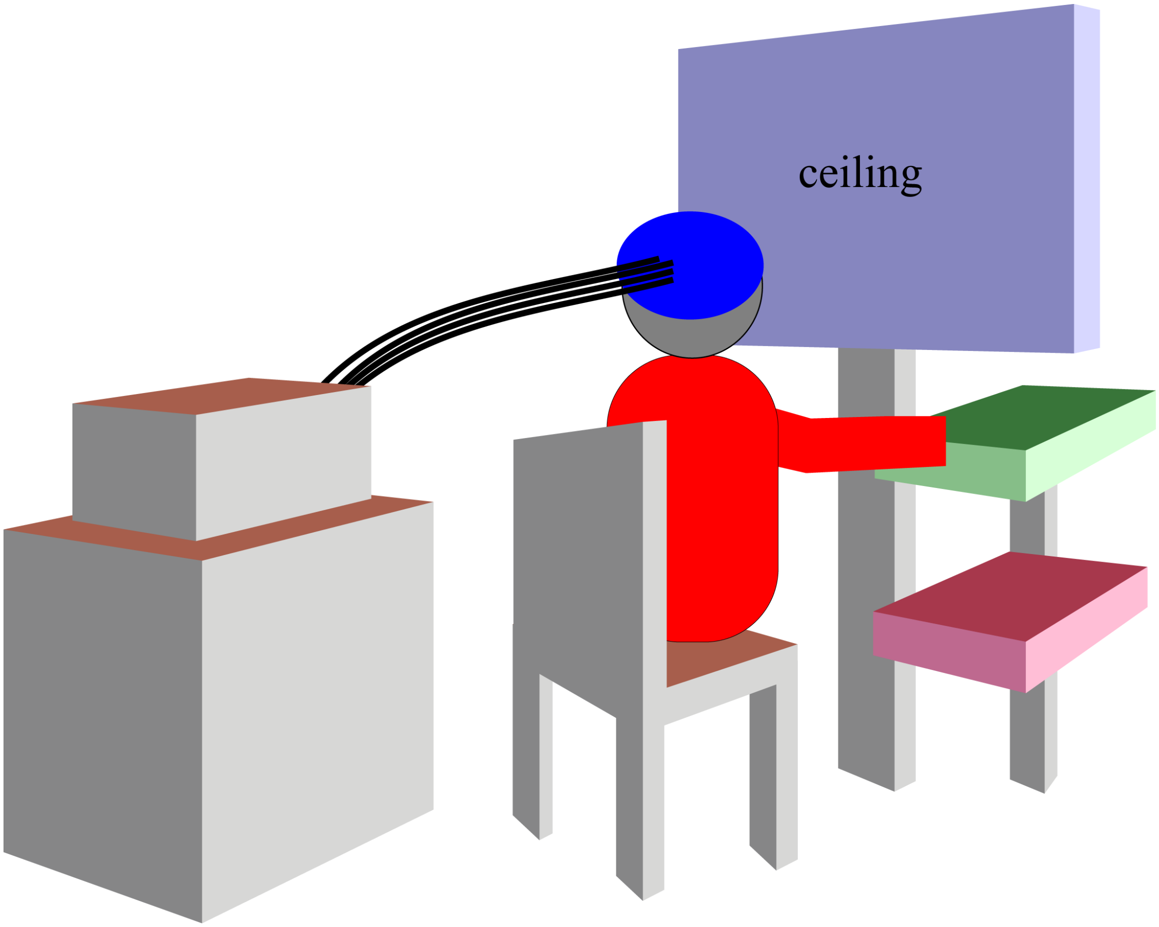

Supplement: Supplementary file 1 [file Image1.PDF]
